# MiR-27a-3p Targets GLP1R to Regulate Differentiation, Autophagy, and Release of Inflammatory Factors in Pre-Osteoblasts *via* the AMPK Signaling Pathway

**DOI:** 10.3389/fgene.2021.783352

**Published:** 2022-01-05

**Authors:** Zhi Zeng, Liangyu Fei, Juntao Yang, Jun Zuo, Zelin Huang, Hao Li

**Affiliations:** ^1^ Department of Joint Surgery, The Second Affiliated Hospital, Hengyang Medical School, University of South China, Hengyang, China; ^2^ Department of Nephrology and Rheumatology, The Affiliated Nanhua Hospital, Hengyang Medical School, University of South China, Hengyang, China; ^3^ Department of Hand and Foot Surgery, The Second Affiliated Hospital, Hengyang Medical School, University of South China, Hengyang, China

**Keywords:** MiR-27a-3p, GLP1R, osteoblast, differentiation, autophagy, inflammation

## Abstract

**Objective:** Osteoporosis is caused by the dysregulation of bone homeostasis which is synergistically mediated by osteoclasts and osteoblasts. MiR-27a-3p is a key inhibitor of bone formation. Hence, unearthing the downstream target gene of miR-27a-3p is of great significance to understand the molecular mechanism of osteoporosis.

**Methods:** Bioinformatics analysis was utilized to find the downstream target gene of miR-27a-3p, and dual-luciferase reporter assay was conducted to validate the interplay of miR-27a-3p and GLP1R. Besides, qRT-PCR, Western blot, and enzyme-linked immunosorbent assay (ELISA) were employed to verify the impact of miR-27a-3p on GLP1R expression and the differentiation, autophagy, and inflammatory response of MC3T3-E1 pre-osteoblasts.

**Results:** Dual-luciferase assay validated that miR-27a-3p directly targeted GLP1R. Additionally, posttreatment of MC3T3-E1 cells with miR-27a-3p mimics resulted in a remarkable decrease in expression levels of GLP1R, cell differentiation marker gene, autophagy marker gene, and AMPK. These results indicated that miR-27a-3p targeted GLP1R to inhibit AMPK signal activation and pre-osteoblast differentiation and autophagy, while promoting the release of inflammatory factors.

**Conclusion:** The miR-27a-3p/GLP1R regulatory axis in pre-osteoblasts contributes to understanding the molecular mechanism of osteoporosis.

## Introduction

Osteoporosis is a typical bone disease resulting from the dysregulation of bone homeostasis (Long, 2011), which is manifested by bone loss, bone resorption prevailing over bone formation, and a reduction in bone volume and density (Yu et al., 2014). Primary osteoporosis is characterized by decreases in osteopsathyrosis and bone mineral density (BMD), whose risk factors included advanced age, the lack of sex hormone, and the increase of oxidative stress (Hendrickx et al., 2015). Bone homeostasis is synergistically modulated by osteoblasts and osteoclasts (Lerner et al., 2019), while organic bone formation is mainly mediated by osteoblasts (Tu et al., 2021). Osteoblast is derived from mesenchyme progenitors and bone precursor cells with the action of a series of transcription factors (TFs), finally differentiating into osteocytes (Long, 2011). Hence, further research on the potential mechanism of autophagy and osteocyte differentiation is urgently needed for osteoporosis therapy.

MiRNAs existing in body fluids can regulate bone formation (Bellavia et al., 2019). MiR-27a-3p is 21 nucleotides in length. A previous study found that miR-27a-3p is a pivotal inhibitor of bone formation, which directly targets primary-response gene osterix and dramatically suppresses osterix expression to inhibit pre-osteoblast differentiation (Xu et al., 2020). This work performed bioinformatics analysis and found that miR-27a-3p might regulate GLP1R to inhibit bone formation. GLP1R is a G protein-coupled receptor of GLP-1 (Song et al., 2017) that activates GLP1R to regulate the insulin secretion, thus affecting osteoporosis (Hennen et al., 2016; Baggio and Drucker, 2021). In addition, liraglutide and Exendin-4 (both are GLP-1 analogue) can upregulate GLP1R expression (Iepsen et al., 2015) to accelerate pre-osteoblast proliferation and differentiation (Feng et al., 2016; Hou et al., 2020; Sun et al., 2020). The above studies exhibited that upregulating the GLP1R expression can dramatically increase bone volume and improve bone micro-architecture as well as the anti-osteoporosis ability of organisms. The interplay between miR-27a-3p and GLP1R may be a vital factor in the occurrence of osteoporosis. Hence, this work investigated the molecular mechanism of miR-27a-3p targeting GLP1R and the effect of the regulatory relationship between these two genes on osteoporosis differentiation.

Autophagy is an energy-supply process during cell differentiation (Ryter et al., 2013), which generates degradation substances to be cyclically used as the selective nutrient supply of cell metabolism (Qi et al., 2017). During osteoblast differentiation, autophagy is activated and inhibiting autophagy can suppress the differentiation ability of osteoblasts (Oliver et al., 2012). Chen *et al.* (Chen et al., 2017) found that activating the GLP1R expression can induce autophagy, reduce apoptosis, and accelerate the proliferation and differentiation of cells. Besides, autophagy promotes osteoblast differentiation and exerts a protective effect on osteoblasts (Li X et al., 2020). In addition, studies indicated that activating AMPK induces autophagy and facilitates cellular differentiation (Ran et al., 2020; Zhang et al., 2020), whereas inhibiting autophagy reveres the effect of AMPK on osteoblast differentiation (Li et al., 2018). Hence, we speculated that miR-27a-3p is also capable of targeting GLP1R and mediate the AMPK signaling pathway to affect osteoblast autophagy and differentiation, thus influencing bone formation.

The dysregulation of inflammation inhibits bone formation and increases bone resorption. The interaction between osteoblasts and inflammatory cells is critical for bone formation, renewal, and remodeling (Mountziaris et al., 2011). Continuous interactions between mononuclear macrophage-osteoclast lineages release cytokines, chemokines, and other factors and accelerate the repair of mesenchymal stem cell-osteoblast lineage, elimination of proinflammatory activity, and remodeling of normal tissue (Kumagai et al., 2008; Mountziaris et al., 2011; Thwaites et al., 2014). Due to the great impact of inflammation and stress on the occurrence of osteoporosis, we further investigated whether miR-27a-3p-targeting-GLP1R is implicated in inflammatory response. Further, we validated that the regulatory relationship between GLP1R and miR-27a-3p influenced osteoporosis.

Our study introduced bioinformatics analyses to find out downstream mRNAs of miR-27a-3p. Afterward, this work studied the binding between GLP1R and miR-27a-3p, as well as the influences of miR-27a-3p targeting GLP1R on osteoblast differentiation, autophagy, and the release of inflammatory factors (IFs). The research on the regulation of miR-27a-3p-GLP1R is helpful to our understanding of the molecular mechanism of osteoporosis, thus contributing to finding the therapeutic target underlying this disease.

## Materials and Methods

### Cell Culture

MC3T3-E1 pre-osteoblasts (BNCC331990, BeNa Culture Collection, Beijing, China) were used for research on osteoblast differentiation. The cells were stored in minimum essential medium-α (MEM-α) supplemented with 1% penicillin–streptomycin (PS) double antibody and 10% fetal bovine serum (FBS). Thereafter, the cells were cultured in an incubator under standard conditions.

### RNA Oligonucleotide Synthesis and Transfection Assays

The oligonucleotides in Table 1 were bought from Sangon Biotech Co., Ltd. (Shanghai, China). GLP1R cDNA was inserted into EcoR I-Hind III (Addgene, Inc., Watertown, MA, USA) sites of the Prk5 vector, and then the oe-GLP1R overexpression vector was obtained. The PCR amplifier of GLP1R cDNA was as follows: forward 5′-GAA​TTC​ATC​AGT​CTG​CGC​ACG​CGG​TTC​CGC-3′; backward 5′-AAG​CTT​GAC​AGT​AGA​CAC​AGA​CTT​TTA​TTT-3′.

**TABLE 1:** Synthesis of miR-27a-3p-related oligonucleotide.

Gene	Sequence 5′-3′
miR-27a-3p mimics	5′-UUC​ACA​GUG​GCU​AAG​UUC​CGC-3′
Anti-miR-27a-3p	5′-GCG​GAA​CUU​AGC​CAC​UGU​GAA-3′
miR-C (control)	5′-UUCUCCGAA CGUGUCACGUTT-3′

A total of 100 nM oligonucleotides and 10 ng oe-GLP1R vectors were taken and respectively transfected into MC3T3-E1 pre-osteoblasts with the Lipofectamine^®^ 2000 Transfection Kit (Invitrogen, Thermo Fisher Scientific, Inc., Waltham, MA, USA). 48 h later, cells were collected, and corresponding RNA and protein expression in cells was measured.

### qRT-PCR Assay

MC3T3-E1 cells were cultured in MEM-α containing 10% FBS for 72 h, and then total RNA was isolated in line with the conventional TRIzol method (Invitrogen, Thermo Fisher Scientific, Inc.). Thereafter, the RNA concentration was gauged, and the RNA sample was diluted to a concentration of 300 ng/μl. Finally, the samples were separated into 3 Eppendorf (EP) tubes and subjected to reverse transcription utilizing the reverse transcription kit (Takara, RR037A, Mountain View, CA, USA).

The quantitative analysis of miR-27a-3p: self-designed stem-loop primer miR-27a-3p RT was used for reverse transcription. The qRT-PCR Kit (Takara, RR820A) was applied to quantitatively analyze miR-27a-3p, using U6 as an internal reference.

Quantitative analysis on related encoding genes involved GLP1R quantitative expression analysis. Osteoblast differentiation marker genes (DMGs) included Runx2, Col1α1 (collagen-1), osteocalcin (OCN), alkaline phosphatase (ALP), and bone sialoprotein (BSP). Autophagy marker genes (AMGs) included BCN1 (ATG7), ATG5, and LC3-II. Inflammatory factor marker genes (IFMFs) included TNFα, interleukin-6 (IL-6), and interleukin-1 (IL-1). β-Actin was used as an internal reference.

The Real-Time PCR System (Applied Biosystems, Foster City, CA, USA) was employed to perform qRT-PCR analysis on each sample 3 times. The conditions for qRT-PCR were initial polymerase activation at 95°C for 2 min followed by 40 cycles of degeneration at 95°C for 1 s and annealing at 60°C for 20 s.

The relative expression levels of genes were calculated using the 2^−ΔΔcq^ method. β-Actin and U6 were respectively internal references of miR-27a-3p and other genes. Primer sequences are shown in Table 2.

**TABLE 2:** qRT-PCR primers.

Genes	Forward primers	Backward primers
miR-27a-3p	5′-GCG​GGC​GTT​CAC​AGT​GGC​TA-3′	5′-CAG​TGC​AGG​GTC​CGA​GGT-3′
U6	5′-CTC​GCT​TCG​GCA​GCA​CA-3′	5′-AAC​GCT​TCA​CGA​ATT​TGC​GT-3′
Runx2	5′-ATG​ATG​ACA​CTG​CCA​CCT​CTG​AC-3′	5′-AACTGC CTG​GGG​TCT​GAA​AAA​GG-3′
ALP	5′-TGA​CCT​TCT​CTC​CTC​CAT​CC-3′	5′-CTT​CCT​GGG​AGT​CTC​ATC​CT-3′
OCN	5′-TGC​TTG​TGA​CGA​GCT​ATC​AG-3′	5′-GAG​GAC​AGG​GAG​GAT​CAA​GT-3′
BSP	5′-AAG​CAG​CAC​CGT​TGA​GTA​TGG-3′	5′-CCT​TGT​AGT​AGC​TGT​ATT​CAT​CCT​C-3′
Col1α1	5′-GCA​ACA​GTC​GCT​TCA​CCT​ACA-3′	5′-CAA​TGT​CCA​AGG​GAG​CCA​CAT-3′
GLP1R	5′-GGG​CCA​GTA​GTG​TGC​TAC​AA-3′	5′-CTT​CAC​ACT​CCG​ACA​GGT​CC-3′
β-actin	5′-CGT​GAC​ATT​AAG​GAG​AAG​CTG-3′	5′- CTA​GAA​GCA​TTT​GCG​GTG​GAC -3′
ATG7	5′-GTT​GCC​GTT​ATA​CTG​TTC​T-3′	5′-TTT​CCA​CCT​CTT​CTT​TGA-3′
ATG5	5′-AAA​GAT​GTG​CTT​CGA​GAT​GTG​T-3′	5′-CAC​TTT​GTC​AGT​TAC​CAA​CGT​CA-3′
LC3	5′-GAC​GGC​TTC​CTG​TAC​ATG​GTT​T-3′	5′-TGG​AGT​CTT​ACA​CAG​CCA​TTG​C-3′
IL-1	5′-GCT​CTG​CCA​TTG​ACC​ATC​TTT​C-3′	5′-CTG​TTA​CTG​CCA​CCA​CAT​TCT​CC-3′
IL-6	5′-CCA​ATT​TCC​AAT​GCT​CTC​CT-3′	5′-ACC​ACA​GTG​AGG​AAT​GTC​CA-3′
TNFα	5′-GAC​ATC​ACT​GGA​GTT​TCC​CCT-3′	5′-CCC​TCC​ATA​CAC​CCG​ACT​TT-3′

### Western Blot Bssay

Total proteins were separated using transfected cells that were cultured for 72 h. Then cells were washed twice using phosphate-buffered saline (PBS). Thereafter, cells were scraped down from the culture dish and collected after centrifugation. Afterward, cells were lysed using 100 µl lysis buffer and incubated on the ice for 15 min, followed by centrifugation at 4°C for 15 min. The cell supernatant was mixed with sodium dodecyl sulfate polyacrylamide gel electrophoresis (SDS-PAGE) sample buffer and heated at 100°C for 5 min. Next, the proteins were isolated in 5%–20% polyacrylamide gel (10 µg total protein/well), and then the isolated proteins were transferred onto a polyvinyl difluoride membrane. Afterward, the membrane underwent a blockage with PBS containing 0.1% Tween-20 and 1% bovine serum albumin (BSA) at usual temperature for 40 min. Then, the membrane was supplemented with primary antibodies for incubation and washed with PBS containing 0.1% Tween-20 for 6 times (5 min/time), followed by a supplement with secondary antibodies for further incubation. After the protein bands were developed and photographed, they were subjected to quantitative analysis using ImageJ software (NIH, Bethesda, MD, USA). β-Actin was the internal reference. Antibodies used in Western blot were purchased from Abcam (Shanghai, China), which included anti-GLP1R (rabbit anti-mouse, monoclonal, cat. no. ab218532), anti-p-AMPK α (rabbit anti-mouse, monoclonal, cat. no. ab133448), anti-AMPKα (rabbit anti-mouse, monoclonal, cat. no. ab32047), anti-β-actin (rabbit anti-mouse, polyclonal, cat. no. ab8227), anti-ATG7 (rabbit anti-mouse, monoclonal, cat. no. ab52472), anti-ATG5 (rabbit anti-mouse, monoclonal, cat. no. ab108327) and anti-LC3B (rabbit anti-mouse, polyclonal, cat. no. ab48394.

### Enzyme-Linked Immunosorbent Assay

ELISA was used to test IL-6, IL-1, and TNFα. The ELISA kit (Andy Gene, Beijing, China) was utilized to study the cell supernatant. A 96-well plate was added with diluent (50 μl), cell supernatant (50 μl), and standard substance (50 μl). Then the plate was washed with cleaning solution (400 μl) and supplemented with human IL-1β or IL-6 conjugate (100 μl). After 2 h of incubation, the plate was supplemented with 100 μl substrate solution and was further incubated for 30 min. In the end, the plate was supplemented with 100 μl termination buffer and optical density (OD) reading at 450 nm was gauged with a microplate reader (Bio-Rad, Hercules, CA, USA).

### Autophagosome Detection

During the autophagy of cells, the LC3 protein progressively transformed into LC3 I protein and then transformed into LC3 II protein which gathered on the membrane of autophagosomes. After transiently transfecting the pEGFP-C1-LC3 plasmid (outright purchase), if autophagy occurred, the LC3 protein would transform into LC3 II protein which was then gathered on the membrane of autophagosomes. By photographing LC3 II protein using a confocal laser-scanning microscope, we confirmed the occurrence of autophagy. Afterward, we estimated the intensity of autophagy by counting the number of autophagosomes. The processes of observation on autophagosomes in MC3T3-E1 pre-osteoblasts were as follows. Firstly, MC3T3-E1 pre-osteoblasts were cultured in a tazetta of the confocal laser-scanning microscope. Then, an appropriate amount of pEGFP-C1-LC3 plasmid was transfected into MC3T3-E1 pre-osteoblasts *via* Lipofectamine transfection and the cells were cultured with CO_2_ in an incubator. After 36 h of culture, the medium was removed and the cells were rinsed using sterile PBS, followed by a fixation with 1 ml of 4% paraformaldehyde (PFA) for 15 min. Thereafter, 4% PFA was removed and the cells were washed with sterile PBS again. Next, the cell nucleus was stained with diamidino-2-phenylindole (DAPI) (Sigma-Aldrich, St. Louis, MO, USA). A small quantity of PBS was added to cover the surface of the cells which were then photographed under the confocal laser-scanning microscope.

### Statistical Analysis

The correlation of expression levels between GLP1R and target miRNA was calculated using the Pearson correlation. Data were subjected to Student-*t* test or one-way analysis of variance (ANOVA) using Bonferroni correction equipped with GraphPad Prism 6.0 software. All groups were compared, and *p* < 0.05 indicated a remarkable difference in statistics.

## Results

### High Expression of miR-27a-3p Inhibits Osteoblast Autophagy and Differentiation

**FIGURE 1:**
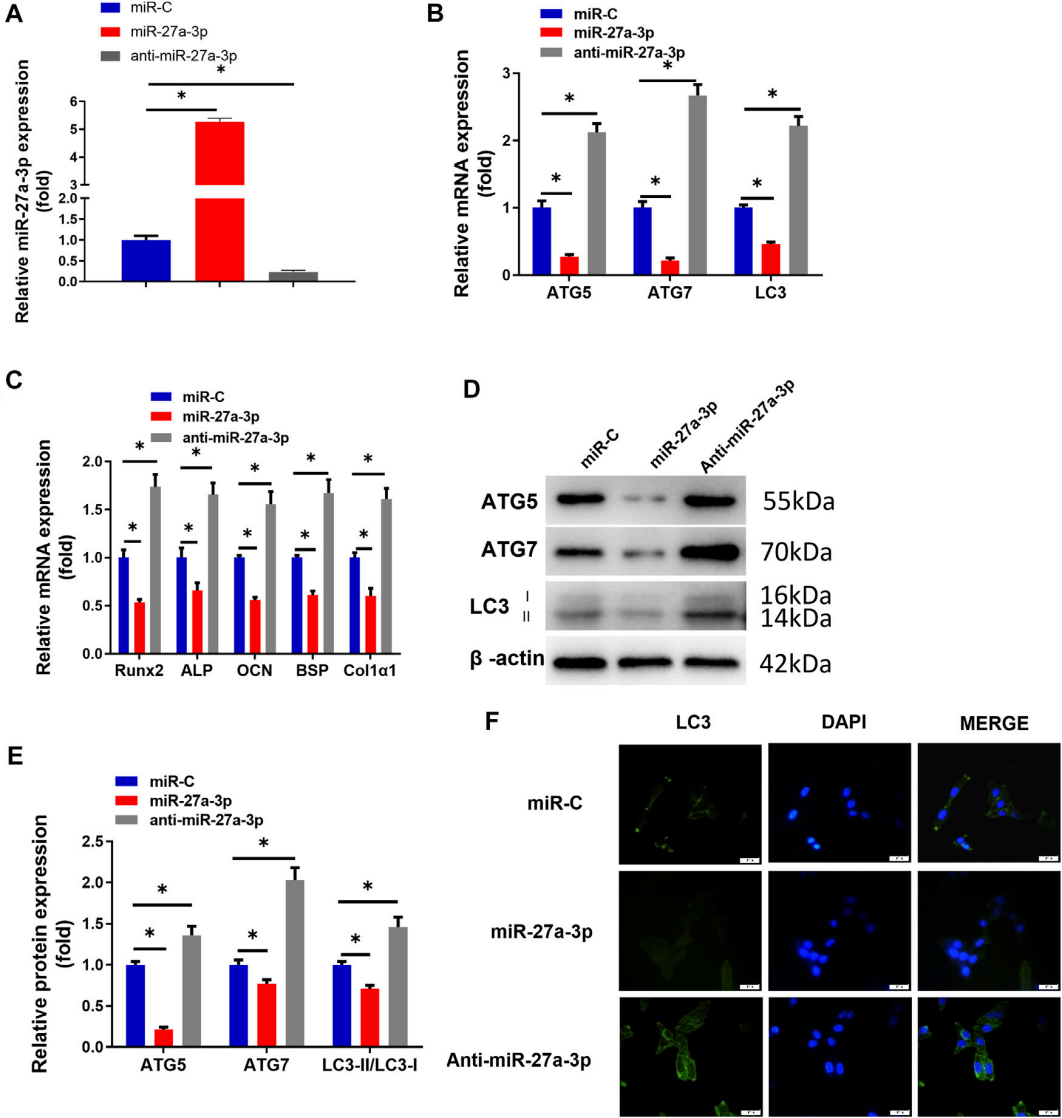
MiR-27a-3p affects osteoblast autophagy and differentiation. **(A)** qRT-PCR validated the transfection efficiency. **(B)** The expression of cell AMGs (LC3, ATG5, and ATG7) in MC3T3-E1 cells. **(C)** The expression of cell DMGs (Runx2, ALP, OCN, BSP, and Col1α1) in MC3T3-E1 cells. **(D, E)** Protein levels of cell AMGs (LC3-I/II, ATG5, and ATG7). **(F)** LC3-II spot images about GFP-LC3 expression in MC3T3-E1 pre-osteoblasts and merged images about GFP-LC3 (green) and DAPI (blue). Figure A–F: MC3T3-E1 pre-osteoblasts were treated with miR-27a-3p mimics, miR-C, and anti-miR-27a-3p respectively. After 24 h of treatment, the cells were subjected to related quantitative detection. *p < 0.05 denotes a significant difference.

Early studies demonstrated that miR-27a-3p is a critical inhibitor of bone formation (Xu et al., 2020). Besides, autophagy facilitates osteoblast differentiation to protect osteoblasts (Li X et al., 2020), while inhibiting autophagy suppresses the differentiation capacity of the cells (Oliver et al., 2012). To explore the impacts of miR-27a-3p on MC3T3-E1 pre-osteoblast autophagy and differentiation, we used miR-27a-3p mimics, anti-miR-27a-3p, and miR-C (the control for the above two) to process MC3T3-E1 pre-osteoblasts and detected the transfection efficiency of miR-27a-3p (Figure 1A). The results exhibited that the expression levels of AMGs (LC3, ATG5, and ATG7) and DMGs (Runx2, ALP, OCN, BSP, and Col1α1) were markedly reduced in the miR-27a-3p mimic treatment group compared to those in the miR-C control group (Figures 1B,C). On the contrary, the expression levels of AMGs (LC3, ATG5, and ATG7), as well as DMGs (Runx2, ALP, OCN, BSP, and Col1α1), were dramatically increased in MC3T3-E1 pre-osteoblasts treated with the anti-miR-27a-3p inhibitor (Figures 1B,C **)**. Similarly, Western blot demonstrated that the expression levels of autophagy marker proteins LC3-II/LC3-I, ATG5, and ATG7 were notably decreased and the number of autophagosomes was noticeably reduced in the miR-27a-3p mimic group (Figures 1D–F). Additionally, the expression of AMGs (LC3-II/LC3-I, ATG5, and ATG7), as well as the number of autophagosomes, was remarkably increased after cells were treated with anti-miR-27a-3p **(** Figures 1D–F). Overall, the overexpression of miR-27a-3p inhibited cellular autophagy and differentiation whereas suppressing this gene showed the opposite effect.

### MiR-27a-3p Downregulates GLP1R Expression

**FIGURE 2:**
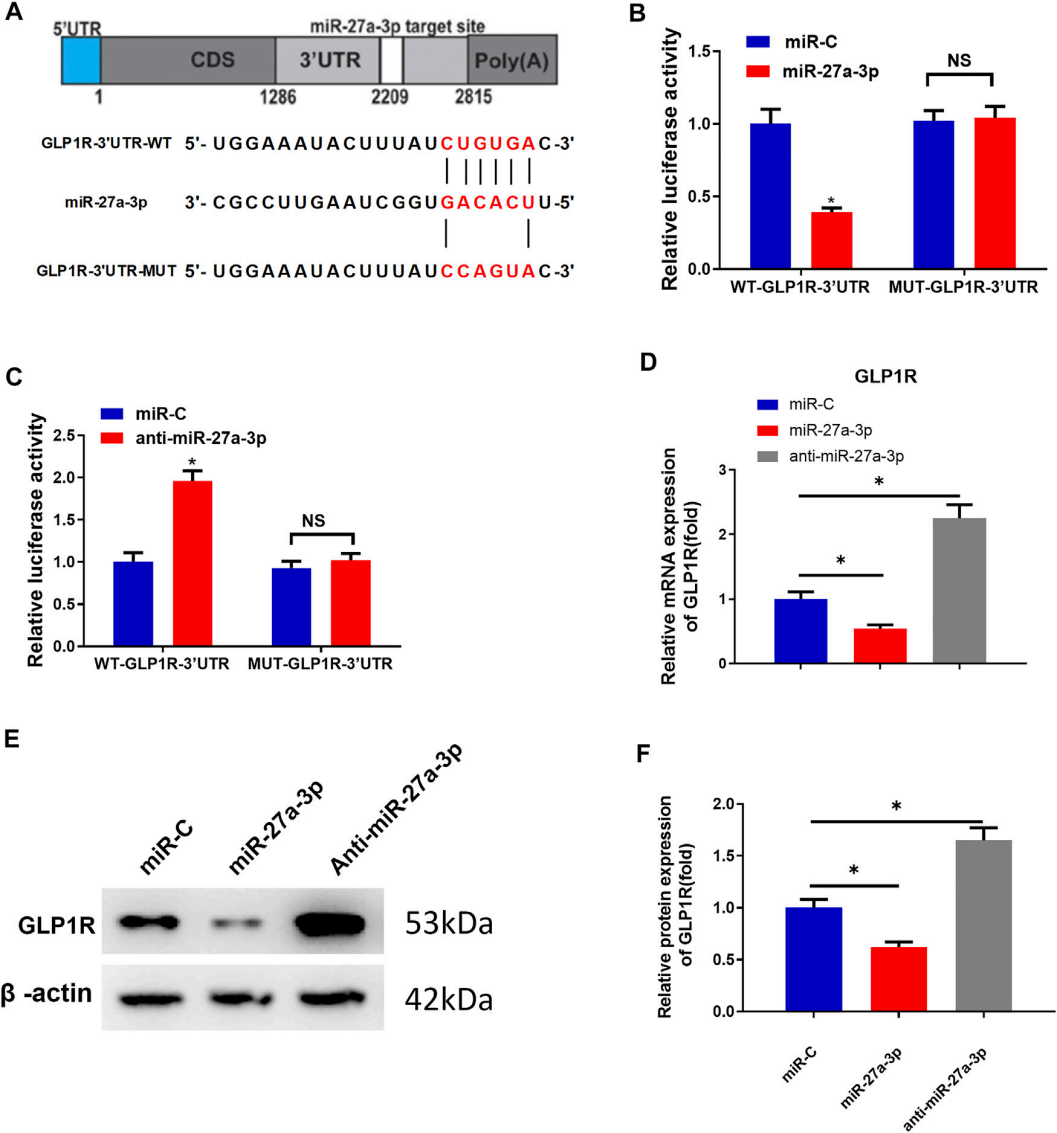
MiR-27a-3p downregulates GLP1R expression. **(A)** Schematic plot about miR-27a-3p targeting the 3′ untranslated region of GLP1R and construction of dual luciferase. **(B)** Outcome of dual-luciferase assay for MC3T3-E cells treated with miR-27a-3p mimics. **(C)** Result of dual-luciferase assay for MC3T3-E1 cells treated with anti-miR-27a-3p. **(D)** GLP1R expression was examined. **(E, F)** Western blot was utilized to measure GLP1R expression. *p < 0.05 indicates a remarkable difference. NS: non-significant difference.

GLP1R as a target of miR-27a-3p is also a pre-proliferation factor for osteoblasts and participates in metabolisms associated with bone formation (Hou et al., 2020; Baggio and Drucker, 2021; Iepsen et al., 2015). To predict whether GLP1R could be targeted by miR-27a-3p, we utilized the starBase website to predict the miRNA–mRNA binding relationship. As predicted, miR-27a-3p directly bound the 3′-UTR of GLP1R (Figure 2A). Mature miR-27a-3p included 21 nucleotide sequences: 5′-UUCA​CAGUGG​CUA​AGU​UCC​GC-3′, which could bind with the 3′-UTR of GLP1R (Figure 2A). An early study has reported that overexpressing GLP1R dramatically increases bone mass, improves bone microstructure, and enhances the anti-osteoporosis ability of organisms (Hennen et al., 2016). We therefore deeply investigated the binding relationship between GLP1R and miR-27a-3p.

To validate the molecular mechanism by which miR-27a-3p targets GLP1R, we respectively merged WT-GLP1R-3′UTR and MUT-GLP1R-3′UTR sequences downstream of the reporter gene. The results exhibited that the luciferase activity of WT-GLP1R-3′UTR treated with miR-27a-3p mimics was markedly decreased, suggesting that miR-27a-3p mimics dramatically suppressed the intensity of the luciferase reporter gene expressing WT-GLP1R-3′UTR. However, the luciferase activity of MUT-GLP1R-3′UTR showed no difference, indicating that miR-27a-3p mimics could not suppress the activity of the luciferase reporter gene expressing MUT-GLP1R-3′UTR (Figure 2B). Similarly, the luciferase activity of WT-GLP1R-3′UTR treated with the anti-miR-27a-3p inhibitor was significantly increased, suggesting that the anti-miR-27a-3p inhibitor notably enhanced the activity of the luciferase reporter gene expressing WT-GLP1R-3′UTR. On the contrary, no difference was shown in the luciferase activity of MUT-GLP1R-3′UTR, which exhibited that the anti-miR-27a-3p inhibitor had no enhanced effect on the activity of the luciferase reporter gene expressing MUT-GLP1R-3′UTR (Figure 2C). To further verify the regulatory relationship between miR-27a-3p and GLP1R, MC3T3-E1 pre-osteoblasts were treated with miR-27a-3p mimics, miR-C, and anti-miR-27a-3p. Besides, qRT-PCR and Western blot were conducted to examine the mRNA and protein expressions of GLP1R. Contrary to control group miR-C, GLP1R expression was dramatically diminished in the miR-27a-3p mimic treatment group (Figures 2D–F). However, GLP1R expression was remarkably increased in MC3T3-E1 pre-osteoblasts treated with anti-miR-27a-3p (Figures 2D–F). Taken together, miR-27a-3p directly targeted the 3′UTR of GLP1R and inhibited GLP1R expression.

### Overexpressing GLP1R Accelerates Pre-Osteoblast Differentiation and Autophagy

**FIGURE 3:**
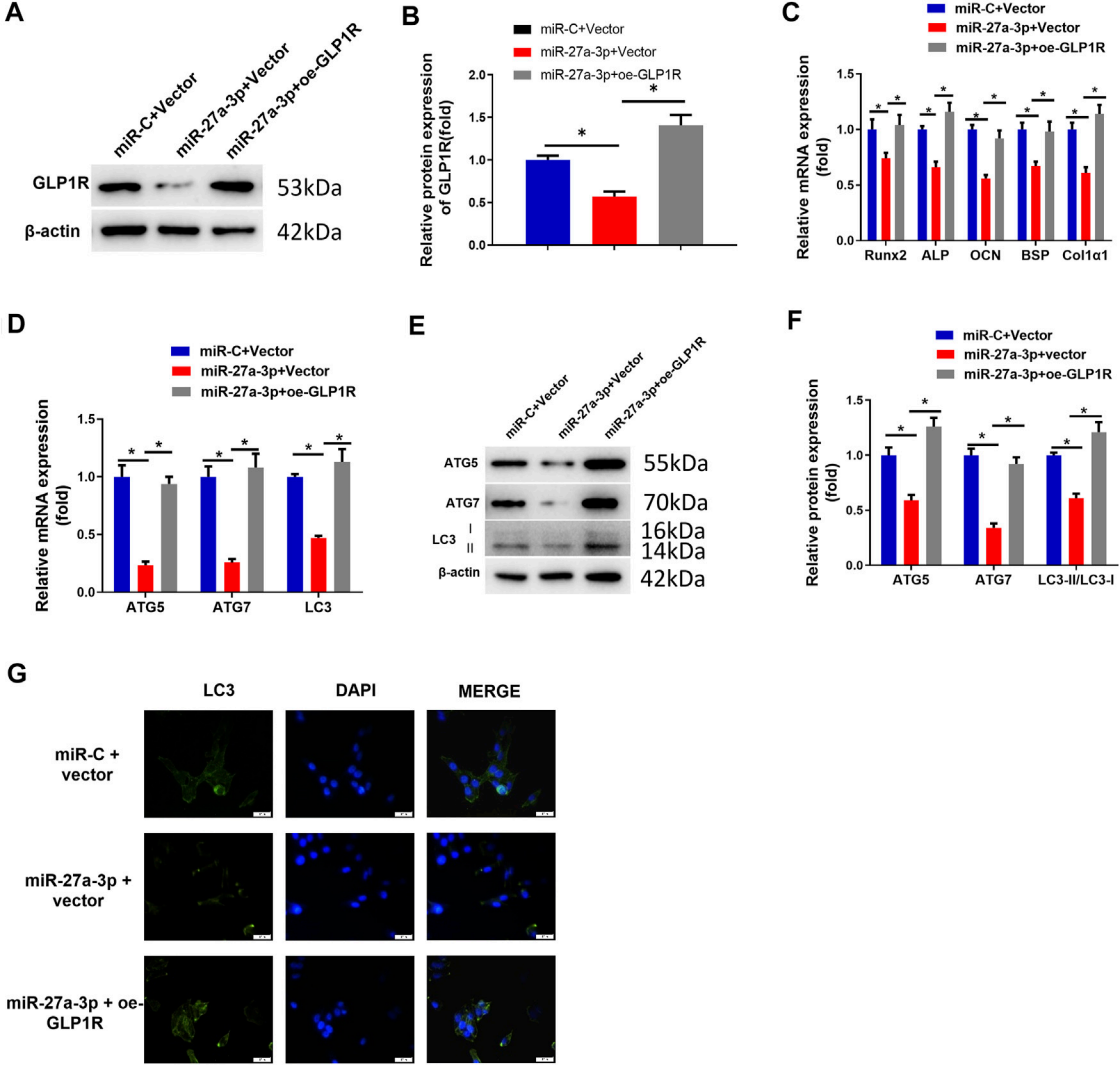
Overexpressing GLP1R facilitates MC3T3-E1 pre-osteoblast differentiation and autophagy. **(A, B)** Protein expression of GLP1R. **(C)** mRNA expression of DMGs (Runx2, ALP, OCN, BSP, and Col1α1) in MC3T3-E1 cells. **(D)** qRT-PCR detected the mRNA expression of AMGs (LC3, ATG5, and ATG7) in MC3T3-E1 cells. **(E, F)** Western blot detected the protein expression of AMGs (LC3, ATG5, and ATG7). **(G)** LC3-II blot images about GFP-LC3 expression in MC3T3-E1 pre-osteoblasts and merged images of GFP-LC3 (green) and DAPI (blue). Figure A–G: Cells were grouped into miR-C + vector, miR-27a-3p + vector, and miR-27a-3p + oe-GLP1R, and then the treated cells were co-transfected into MC3T3-E1 pre-osteoblasts, followed by a quantitative detection after 24 h. *p < 0.05 denotes a remarkable significance.

To investigate whether overexpressing GLP1R could reverse the suppressive impact of miR-27a-3p on osteoblast differentiation and autophagy, we established the GLP1R overexpression cell line (oe-GLP1R). Thereafter, miR-27a-3p mimics and oe-GLP1R were co-transfected into MC3T3-E1 cells. Contrary to the control groups, oe-GLP1R could restore the effect of miR-27a-3p mimics (Figures 3A,B). Besides, the expressions of AMGs (LC3, ATG5, and ATG7) and DMGs (Runx2, ALP, OCN, BSP, and Col1α1) were restored in co-transfected cells (Figures 3C–F). Additionally, there was a marked increase in the number of autophagosomes as well (Figure 3G). The above results exhibited that the overexpression of GLP1R diminished the inhibitory impact of miR-27a-3p on autophagy and osteoblast differentiation. Generally, miR-27a-3p restrained autophagy and osteoblast differentiation through downregulating the GLP1R expression.

### MiR-27a-3p Targets GLP1R to Inhibit the AMPK Signaling Pathway

**FIGURE 4:**
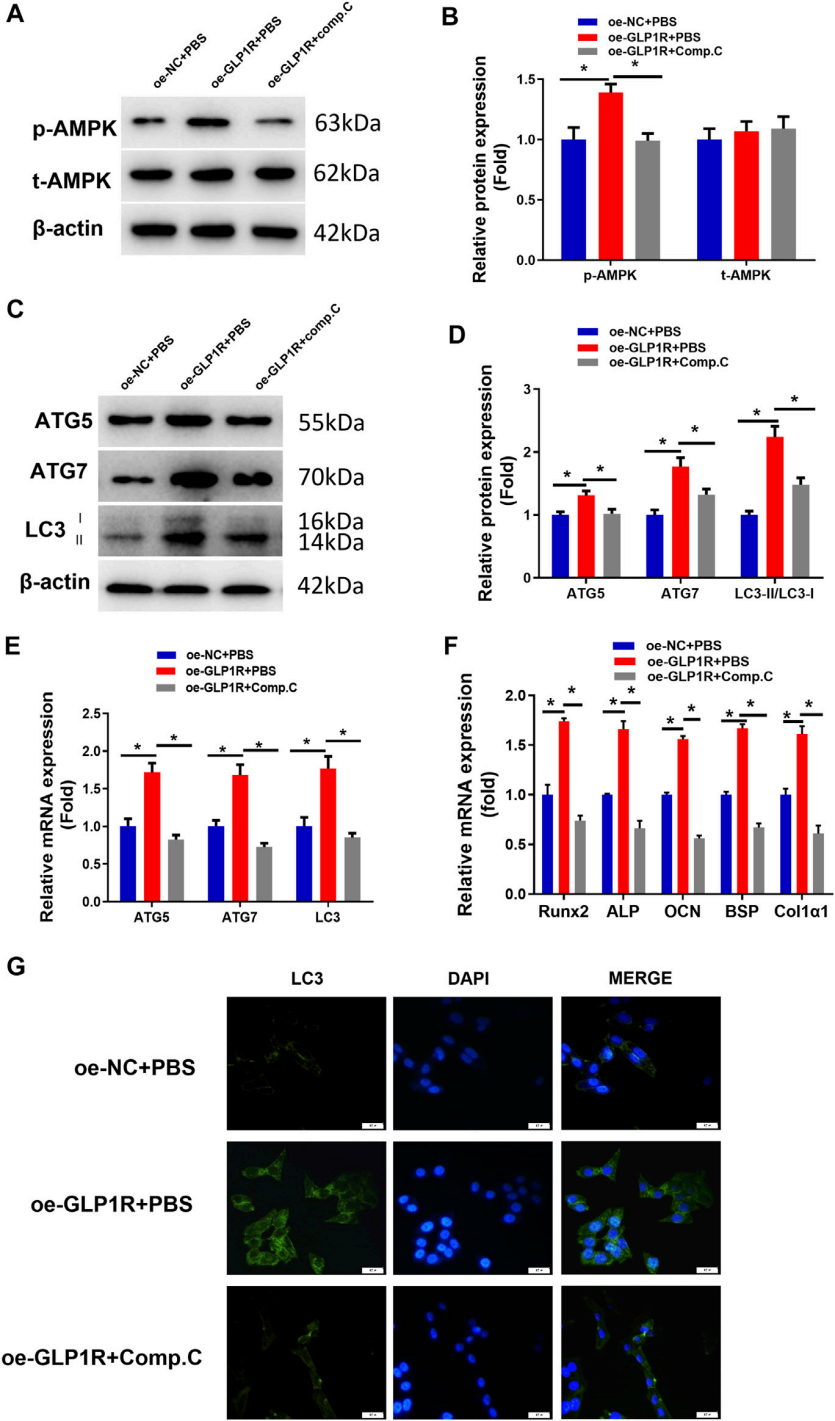
Overexpressing GLP1R activates the AMPK signaling pathway. **(A, B)** Protein levels of AMPK and p-AMPK in MC3T3-E1 cells. **(C, D)** Expression of AMGs (LC3, ATG5, and ATG7) in MC3T3-E1 cells was examined utilizing Western blot. **(E)** Expression of AMGs (LC3, ATG5, and ATG7) in MC3T3-E1 cells. **(F)** Expression of DMGs (Runx2, ALP, OCN, BSP, and Col1α1) in MC3T3-E1 cells was detected using qRT-PCR. **(G)** LC3-II blot images about GFP-LC3 expression in MC3T3-E1 pre-osteoblasts and merged images of GFP-LC3 (green) and DAPI (blue). *p < 0.05 indicates a marked difference.

Studies found that activating AMPK can induce cellular autophagy to accelerate cellular differentiation (Ran et al., 2020; Zhang et al., 2020). Hence, we investigated whether miR-27a-3p targets GLP1R to affect osteoblast autophagy and differentiation by mediating the AMPK signaling pathway. To verify this speculation, we applied compound C (phosphorylation inhibitor) to the oe-GLP1R cell line to figure out whether suppressing AMPK phosphorylation can counteract the enhanced effect of GLP1R overexpression. The assay demonstrated that overexpressing GLP1R significantly enhanced AMPK phosphorylation. However, after the cells were treated with compound C, AMPK phosphorylation was dramatically attenuated (Figures 4A,B). Correspondingly, overexpressing GLP1R markedly increased the expression of AMGs (LC3, ATG5, and ATG7) whereas cells being treated with compound C showed the opposite effect (Figures 4C–E). Meanwhile, the number of autophagosomes was also significantly recovered after the cells were treated with compound C (Figure 4G). Together, suppressing AMPK phosphorylation counteracted the enhanced impact of overexpressing GLP1R on osteoblast autophagy. Additionally, treating with compound C also reduced the expression of DMGs (Runx2, ALP, OCN, BSP, and Col1α1) and reversed the promoted effect of overexpressing GLP1R on osteoblast differentiation (Figure 4F). The above outcomes indicated that suppressing AMPK phosphorylation could counteract the enhanced effect of overexpressing GLP1R on osteoblast autophagy and differentiation. In other words, miR-27a-3p downregulated GLP1R to suppress osteoblast autophagy and differentiation *via* mediating the AMPK signaling pathway.

### MiR-27a-3p Targets GLP1R to Modulate Inflammatory Response

**FIGURE 5:**
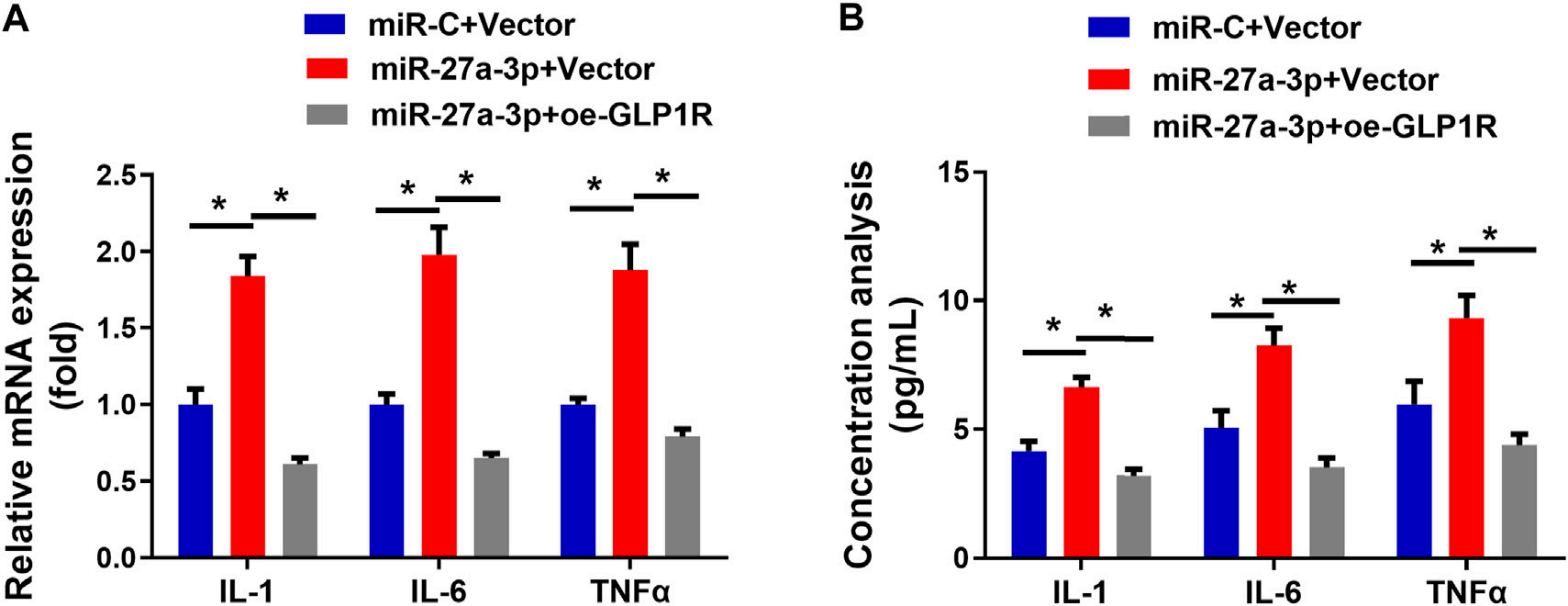
miR-27a-3p impacts on inflammatory response of MC3T3-E1 pre-osteoblasts. **(A)** Levels of IFs (IL-1, IL-6, and TNFα) in MC3T3-E1 pre-osteoblasts. **(B)** The concentration of IFs (IL-1, IL-6, and TNFα) in cell medium. *p < 0.05 denotes a remarkable difference.

Inflammatory dysregulation leads to an increase in bone resorption and a suppression of bone formation (Hendrickx et al., 2015). The interaction between inflammatory cells and osteoblasts is critical for bone formation, repair, and remodeling (Mountziaris et al., 2011). Finally, inflammation inhibits osteoblast differentiation, resulting in osteoporosis. Hence, we aimed to further investigate whether targeting of GLP1R by miR-27a-3p affects inflammatory response (Figure 5). IL-1, IL-6, and TNF-α are probably indicators for bone injury (Kon et al., 2001; Cho et al., 2002). The MiR-27a-3p mimic activated 3 cytokines at the mRNA level which could be attenuated by the oe-GLP1R + miR-27a-3p mimic (Figure 5A). The protein levels also manifested a similar trend (Figure 5B). Hence, we could conclude that miR-27a-3p facilitated the inflammatory response of cells while overexpressing GLP1R could counteract such effect. MiR-27a-3p therefore targeted GLP1R to accelerate the inflammatory response of osteoblasts.

## Discussion

**FIGURE 6:**
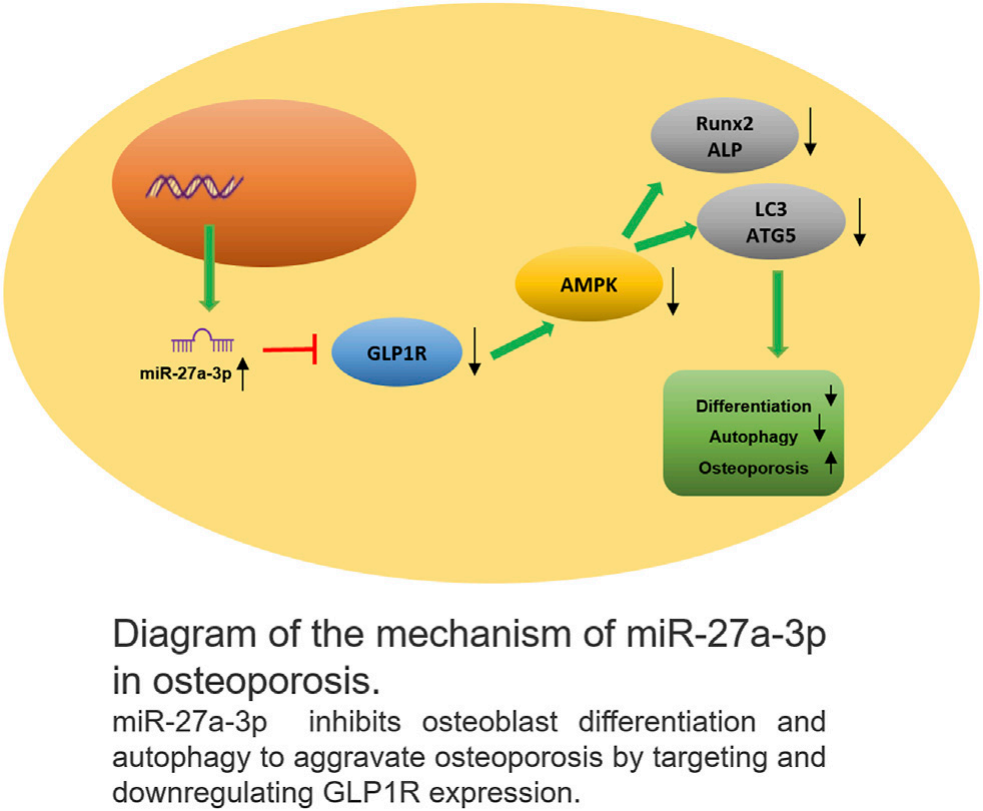
Schematic plot about the action mechanism of miR-27a-3p in osteoporosis MiR-27a-3p downregulated GLP1R to inhibit osteoblast differentiation and autophagy, thus exacerbating osteoporosis.

Our study analyzed and illustrated the relationship between GLP1R and miR-27a-3p in osteoblasts. Firstly, this work validated that miR-27a-3p downregulated GLP1R and that GLP1R mediated the AMPK signaling pathway to modulate osteoblast autophagy and differentiation. Based on the results of previous studies, we proposed an osteoporosis-related regulatory mechanism (Figure 6). In this mechanism, downregulation of GLP1R by miR-27a-3p inhibited AMPK phosphorylation to suppress osteoblast autophagy and differentiation, thus causing osteoporosis.

The aberrant expression of miRNAs in osteoporosis leads to osteoporosis, and miRNAs can thereby serve as potential targets of treatment for osteoporosis. For instance, miRNA-30a-5p upregulates RUNX2 to induce osteoblast differentiation, thus alleviating osteoporosis (Zhang et al., 2019). On the contrary, miR-125a-5p upregulates TNFRSF1B to induce osteoclasts, thereby aggravating this disease (Sun et al., 2019). MiR-27a-3p is a key inhibitor of bone formation, which can target PPARγ and GREM1 to modulate bone formation and influence downstream osterix to suppress osteoblast differentiation (Xu et al., 2020). This work found that miR-27a-3p could affect osteoblast autophagy and differentiation. Despite the effect of miR-27a-3p on bone formation, miRNA does not usually act as an effector molecule to regulate bone formation. Hence, we further studied the downstream target that might be regulated by miRNA. Bioinformatics and molecular assays suggested that GLP1R was a downstream gene of miR-27a-3p. We, therefore, speculated that miR-27a-3p targeted GLP1R to affect the occurrence of osteoporosis.

GLP1R is expressed in multiple organs (Ceccarelli et al., 2013). Current studies reported that GLP1R is associated with the occurrence of osteoporosis. For example, Hansotia *et al.* (Hansotia and Drucker, 2005) pointed out that GLP1R contributes to the treatment of diabetes and osteoporosis. In addition, studies also indicated that GLP-1 activates GLP1R to alleviate osteoporosis (Montes Castillo et al., 2019; Schiellerup et al., 2019). Meng *et al.* (Meng et al., 2016) found that Exendin-4 can activate GLP1R expression to ameliorate osteoporosis *via* the PKA/β-catenin signaling pathway. After verifying the regulatory relationship between miR-27a-3p and GLP1R, subsequent cellular function assay showed that miR-27a-3p inhibited GLP1R expression to affect osteoblast autophagy and differentiation, which played a vital role in osteoporosis. Previous studies found that liraglutide can activate GLP1R expression to enhance cell autophagy and differentiation *via* the AMPK signaling pathway (Kong et al., 2018). We speculated that GLP1R could also affect osteoblast autophagy. Hence, we conducted cellular assay and validated that GLP1R increased the expression of autophagy genes like ATG5, ATG7, and LC3 and activated AMPK phosphorylation. In sum, we found that GLP1R regulated the activity and autophagy of the AMPK signaling pathway to influence the occurrence of osteoporosis.

The damage of bone induces acute inflammation to affect the repair of local bone. Cross talk between cells related to bone healing and inflammatory cells is a crucial factor of bone formation, repair, and remodeling (Loi et al., 2016). We also examined the expression of IFs related to osteoblast inflammation, suggesting that miR-27a-3p facilitated the inflammatory response of MC3T3-E1 pre-osteoblasts. Inflammation is an instantaneous reaction, which plays a pivotal part in the healing of fractures and bone damage. Regenerative inflammation of bone tissue contributes to the repair of bone. On the contrary, destructive inflammation leads to the resorption of bone tissue, and dysregulation of inflammation increases bone resorption and suppresses bone formation, causing osteoporosis (Mountziaris et al., 2011). MiRNA also exerts a significant effect on the regulation of bone inflammation. For example, Li *et al.* (Li Z et al., 2020) pointed out that miR-29a-3p regulates the inflammatory response of osteoclast to affect the occurrence of osteoporosis. The results of our study demonstrated that miR-27a-3p can act as a suppressor of bone formation and destroy the balance of inflammation in bone tissue. Altogether, we assumed that miR-27a-3p targeted GLP1R to regulate inflammatory stress, thereby inhibiting bone formation.

Dysregulation of osteoclasts also contributes to bone loss (Andreev et al., 2020). One study has pointed out abnormally expressed miR-27a-3p during osteoclast formation (Ma et al., 2016). Therefore, we assumed that miR-27a-3p/GLP1R might be implicated in osteoporosis *via* influencing osteoclast behaviors and plan to evaluate such axis in osteoclasts.

MiR-27a-3p downregulated GLP1R to suppress osteoblast autophagy and differentiation *via* mediating the AMPK signaling pathway. MiR-27a-3p targeted GLP1R to accelerate inflammatory response of osteoblasts. Besides, targeting GLP1R by miR-27a-3p affected the inflammatory stress of osteoblasts. Our study still has limitations. For example, although AMPK signaling in inflammation and ROS production has been elaborated (Pilon et al., 2004; Wen et al., 2011), we failed to identify whether they happened during miR-27a-3p-induced bone loss. Also, we did not verify the effect of miR-27a-3p on osteoporosis *in vivo*, which will be a subject of our further study. If we intend to develop osteoporosis drugs based on our results, we still need to perform clinical and animal experiments to verify the results in future studies.

## Data Availability

The original contributions presented in the study are included in the article/Supplementary Material, further inquiries can be directed to the corresponding author.
