# Supplementary material for: MiR-27a-3p Targets GLP1R to Regulate Differentiation, Autophagy, and Release of Inflammatory Factors in Pre-Osteoblasts via the AMPK Signaling Pathway
Source: Front Genet. 2022 Jan 5;12:783352. doi: 10.3389/fgene.2021.783352 (PMC8766720; doi:10.3389/fgene.2021.783352)
Supplement: Supplementary file 1 [file Table1.DOCX]

**Table 1** **Synthesis of miR-27a-3p-related oligonucleotide**

| Gene | Sequence 5’-3’ |
| --- | --- |
| miR-27a-3p mimics | 5’-UUCACAGUGGCUAAGUUCCGC-3’ |
| anti-miR-27a-3p | 5’-GCGGAACUUAGCCACUGUGAA-3’ |
| miR-C (control) | 5’-UUCUCCGAA CGUGUCACGUTT-3’ |
