# Supplementary material for: MiR-27a-3p Targets GLP1R to Regulate Differentiation, Autophagy, and Release of Inflammatory Factors in Pre-Osteoblasts via the AMPK Signaling Pathway
Source: Front Genet. 2022 Jan 5;12:783352. doi: 10.3389/fgene.2021.783352 (PMC8766720; doi:10.3389/fgene.2021.783352)
Supplement: Supplementary file 2 [file Table2.DOCX]

**Table 2 qRT-PCR primers**

| **Genes** | **Forward primers** | **Backward primers** |
| --- | --- | --- |
| miR-27a-3p | 5’-GCGGGCGTTCACAGTGGCTA -3’ | 5’-CAGTGCAGGGTCCGAGGT -3’ |
| U6 | 5’-CTCGCTTCGGCAGCACA- 3’ | 5’-AACGCTTCACGAATTTGCGT-3’ |
| Runx2 | 5’-ATGATGACACTGCCACCTCTGAC-3’ | 5’-AACTGC CTGGGGTCTGAAAAAGG-3’ |
| ALP | 5’-TGACCTTCTCTCCTCCATCC-3’ | 5’-CTTCCTGGGAGTCTCATCCT-3’ |
| OCN | 5’-TGCTTGTGACGAGCTATCAG-3’ | 5’-GAGGACAGGGAGGATCAAGT-3’ |
| BSP | 5’-AAGCAGCACCGTTGAGTATGG-3’ | 5’-CCTTGTAGTAGCTGTATTCATCCTC-3’ |
| Col1α1 | 5’-GCAACAGTCGCTTCACCTACA-3’ | 5’-CAATGTCCAAGGGAGCCACAT-3’ |
| GLP1R | 5’-GGGCCAGTAGTGTGCTACAA-3’ | 5’-CTTCACACTCCGACAGGTCC-3’ |
| β-actin | 5’- CGTGACATTAAGGAGAAGCTG -3’ | 5’- CTAGAAGCATTTGCGGTGGAC -3’ |
| ATG7 | 5’-GTTGCCGTTATACTGTTCT-3’ | 5’-TTTCCACCTCTTCTTTGA-3’ |
| ATG5 | 5’-AAAGATGTGCTTCGAGATGTGT-3’ | 5’-CACTTTGTCAGTTACCAACGTCA-3’ |
| LC3 | 5’-GACGGCTTCCTGTACATGGTTT-3’ | 5’-TGGAGTCTTACACAGCCATTGC-3’ |
| IL-1 | 5’-GCTCTGCCATTGACCATCTTTC-3’ | 5’-CTGTTACTGCCACCACATTCTCC-3’ |
| IL-6 | 5’-CCAATTTCCAATGCTCTCCT-3’ | 5’-ACCACAGTGAGGAATGTCCA-3’ |
| TNFα | 5’-GACATCACTGGAGTTTCCCCT-3’ | 5’-CCCTCCATACACCCGACTTT-3’ |
